# A Systematic Review of the Clinical Efficacy of Micro-Focused Ultrasound Treatment for Skin Rejuvenation and Tightening

**DOI:** 10.7759/cureus.20163

**Published:** 2021-12-04

**Authors:** Ubaid Khan, Nabiha Khalid

**Affiliations:** 1 Medicine, Mayo Hospital Lahore, Lahore, PAK; 2 Biochemistry, University of Gujrat, Gujrat, PAK

**Keywords:** tightening, non-invasive treatment method, facial laxity, skin rejuvenation, micro-focused ultrasound

## Abstract

The demand for non-invasive skin-tightening techniques is continuously on the rise, as now numerous patients seek safe and effective alternative body, neck, and facial aesthetic surgical procedures. Micro-focused ultrasound (MFU) has been recently introduced as a novel energy modality for skin rejuvenation to produce a more significant wound healing response at various levels, including strong collagen remodeling and long-lasting clinical response. This literature study was intended to find the role and efficiency of using micro-focused ultrasound therapy in male and female patients aged 35-65. A total of 139 articles were extracted from the PubMed and Science Direct electronic databases. After a thorough evaluation and following the exclusion and inclusion criteria, 10 full-text articles were relevant to the study. The goal was to analyze and examine the effects and benefits of MFU treatment to improve the skin. In addition, all of the patients were evaluated to report the harms and risks associated with MFU treatment. The literature study results revealed that significant improvements in the overall aesthetics of sagging of the mid and lower face could be accomplished by using a micro-focused ultrasonic treatment plan. Patients report no considerable side effects, and the results were also long-lasting. MFU treatment can activate deeper tissues without causing injury to the epidermis, which sets it apart from all other skin tightening methods. Better improvements rates have been reported by both patients' self-assessment and clinical investigators' evaluation.

## Introduction and background

Surgical lifting procedures have typically been used to treat facial and neck skin sagging and laxity. However, a broad range of nonsurgical procedures has emerged as an alternative to surgery over the last decade. Through the administration of controlled dermal heating, treatments, including fractional and ablative laser skin resurfacing and radiofrequency (RF), offer varying tissue tightening levels.

Traditional ablative laser skin rejuvenation is associated with a lengthy healing period and the possibility of delayed dyspigmentation [1]. Additionally, heat penetration up to 2-4 mm into the skin is needed to achieve minor skin tightening produced by RF devices. Thus, it promotes healing wounds and neocollagenesis without causing epidermal damage or clinical recovery [2]. When compared to ablative laser skin rejuvenating or surgical face lifting procedures, the advantages of this method are indistinct: minimal downtime, relative safety for usage on non-facial areas and skin color, and a favorable side effect profile [3]. Unfortunately, less intrusive procedures have a history of poor efficacy, uneven clinical outcome, and a shorter-lasting tightening effect.

Studies reported that the acoustical energy of high-intensity focused ultrasound (HIFU) is considered to penetrate tissue far deeper than laser or RF radiation. And these have recently been developed to treat subcutaneous lipolysis [4]. Ultrasound waves penetrate tissue, causing molecules to vibrate at just the focus of the beam. So at the focal point of the beam, friction among tissue molecules creates thermal damage. The depth of penetration is determined by the frequency of the waves, with higher frequency waves generating a lower focal injury zone. In contrast, shallow frequency waves generate focal thermal injury zones (TIZs) across deeper layers [5].

Micro-focused ultrasound (MFUS) was introduced in 2009 to offer precision-focused thermal injury zones at therapeutic depths larger than those available with the previous technologies [6]. In addition, it is capable of delivering deep heat energy into the superficial dermis and subdermal connective tissue at tissue planes to induce more extensive collagen remodeling [7]. That's why the MFUS device may be ideally adapted to treat the problem of skin laxity.

MFUS, in opposed to HIFU, offers more accurate energy delivery due to advancements in the system that better meet the needs of skin laxity [8]. Previous studies indicated that variations in small durations of pulse combined with greater frequency transducers enable MFUS to produce specific areas of coagulative necrosis, known as TIZs, for transcutaneous therapy. Every TIZ is precisely concentrated at a specific depth and heated with smaller pulses (150 ms) to induce shallow zones (1 mm^3^) of coagulative necrosis at the location. However, the superficial layers and surrounding tissue mostly remain unaffected. The thermal harm is limited, much like a laser pulse, by maintaining the pulse duration small [8-9].

Moreover, as long as the energy supplied does not exceed the frequency and focal depth radiated for a particular transducer, the epidermal surface is kept undisturbed. This thus removes the requirement for shallow cooling and expediting the retrieval procedure, as healing proceeds quickly from untreated neighboring tissue [9-10]. The MFUS approach can reach deeper into tissue than its nonsurgical counterparts. Engaging the superficial musculoaponeurotic system (SMAS) achieves more excellent tissue tightness and benefits that last longer. The SMAS runs superficially to engage with the dermis and goes deep into the subcutaneous fat. It surrounds the muscles of facial expression and lies deeper in the subcutaneous fat. The SMAS layer, like the dermal layer of the skin, is made up of collagen and elastic fibers, but it has a more lasting retention ability and compared to the skin alone, shows less delayed relaxation following lifting procedures [11]. As a result, the SMAS is a reported great target for non-invasive skin tightening.

MFU causes an immediate contraction of denatured collagen through heat stimulation, triggers neocollagenesis, and collagen remodeling with subsequent skin tightening. It accomplishes this by establishing tiny, precisely regulated thermal coagulation sites in the mid to deep reticular dermis, all the way up to the superficial muscular aponeurotic system (SMAS) [12]. The Food and Drug Administration (FDA) has approved using an MFU device (Ulthera, Ulthera Inc., Mesa, Arizona) to raise the tissues in the brow, neck, and submentum without invasive surgery. The Ulthera system is an MFU device that is used for this purpose. Micro-focused ultrasound (MFU) therapy is combined with high-resolution ultrasound imaging to transfer energy to exact depths (up to 5 mm) inside the skin's dermal layers and SMAS while protecting the epidermal layers. The Ulthera system encourages the introduction of new tissue as well as collagen and elastin remodeling by modulating thermally induced tissue contraction and a wound-healing response. The heat is contained within small focal patches of the dermis, avoiding the epidermis and surrounding tissue [13].

## Review

Methodology

The inclusion and exclusion criteria of this study are listed in Table 1 [14].

**Table 1:** Inclusion and Exclusion Criteria

Inclusion	Exclusion
Males and Females	Pregnant or Lactating Females
Age: 35-65 years	Excessive Sub-Cutaneous Fat on the Cheeks
Good Health Condition	Cystic or Severe Facial Acne
Individuals Looking for Face Skin Rejuvenation	Any Local or Active Systematic Skin Disease That Can Hinder the Wound-Healing Process
Individuals Looking for Lifted and Tightened Cheeks,	Open Lesions and Wounds in the Targeted Treatment Area
Individuals Wanting to Improve Skin Laxity	Individuals With Mental illness
Naïve to Minimal Invasive or Nonsurgical Treatments	History of Undergoing Cosmetic Treatments in the Targeted Facial Area
Willing to Provide Informed Consent	Individuals Unable to Provide Informed Consent
Willing to Attend Follow-up Visits	Individuals Unable to Understand the Protocol

Search strategies

For the collection of data, the search was performed using the PubMed and Science Direct electronic databases. The Google Scholar platform was used for article downloading and citation. The research papers were selected from the year 2013 to 2021. To search required studies and articles, MeSH terms were employed for PubMed to determine the best and optimal suited keywords. The focus keywords for search purposes were "Micro Focused Ultrasound," "Skin Rejuvenation," and "Skin Laxity Treatment." Only human studies were included, and any clinical trials on animals and other non-humanized models were excluded. While searching from Science Direct, the search method was optimized to select only those papers that contain the required keywords in the title or the abstract. The same technique opted for the PubMed search. Furthermore, the option to exclude non-humanized trials was not available on Science Direct; each publication was manually verified to ensure that only human studies were included for review writing purposes. The articles in the Bibliography section of selected articles were also searched for full text.

Result of the data search

**Figure 1:**
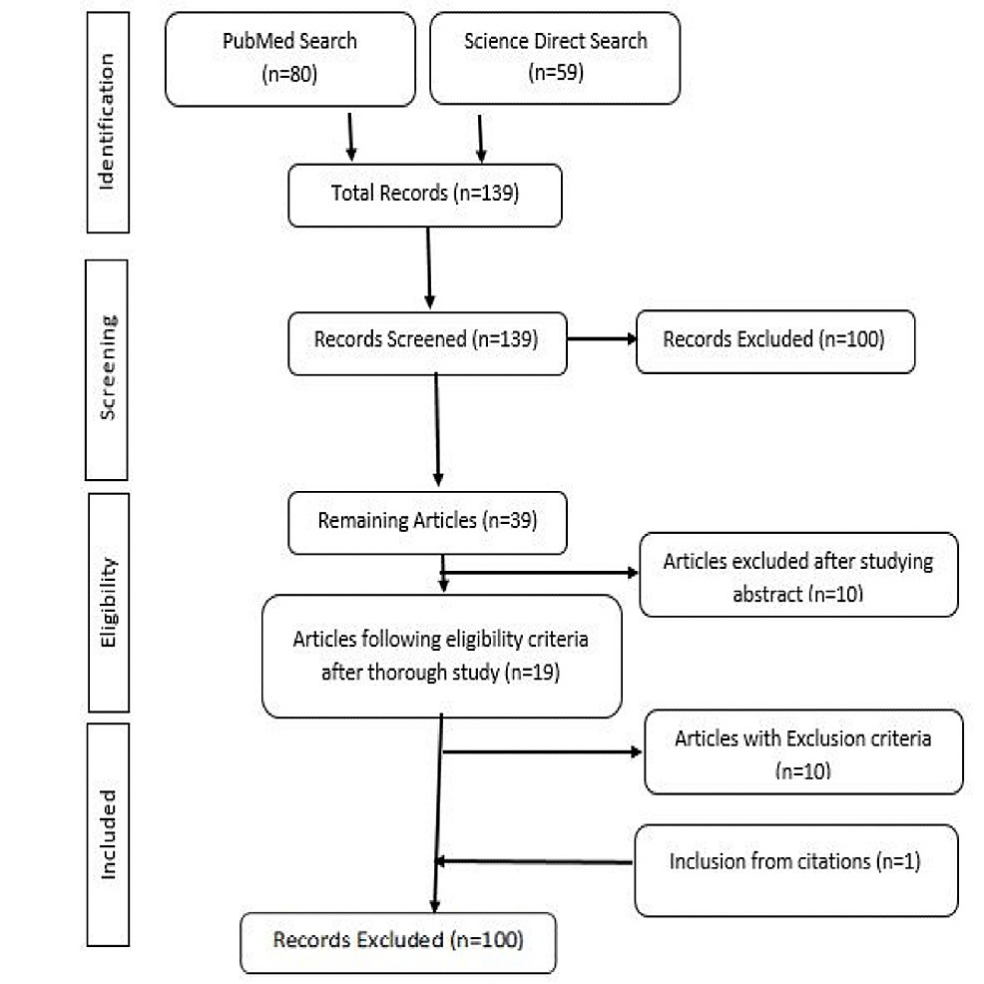
Search Strategy for Data Collection on the Use of Micro-Focused Ultrasound Treatment for Skin Improvement

The search strategy on micro-focused ultrasound for skin improvement recommended 80 articles on PubMed and 59 papers on Science Direct. The total number of articles thus found was 139. After cross-checking and comparing the titles of articles, irrelevant and repeated articles were excluded from the search. So from a total of 139 articles, 100 were found to be irrelevant to the targeted search and thus were eliminated. The remaining number of articles after this step was 39. After studying the abstract of these articles, 10 were discarded. The remaining 19 articles were further thoroughly studied and investigated to find whether they meet the inclusion criteria or not. Ten of these articles didn't follow the inclusion criteria, so they were also eliminated. Finally, one article was further selected after cross-checking the bibliography of already selected articles and was included too. In the end, 10 articles that entirely meet the inclusion criteria were finally selected for review writing (Figure 1) [14].

Results and discussion

Murad Alam et al. conducted the initial clinical research to determine if MFU-V might raise the brow during the treatment of the whole neck and face through the application of micro-focused ultrasound [14]. MFU-V was applied on the temples, submental region, cheeks, side of the head, and forehead using three transducers. These were used to emit 4 MHz and 7 MHz thermal energy at focal depths of 4.5 mm and 3.0 mm, respectively. A total of 36 people (34 females) were enrolled for this study, with one subject dropping out and 35 being evaluated. The average age was 44 years old (range 32-62). The evaluators considered 30 of 35 individuals (86%) to have a clinically significant brow-lift 90 days following treatment (P =.00001). As measured by pictures at 90 days, the average change in eyebrow height was 1.7 mm, as per the second primary outcome measure [14].

Several studies have found that using numerous micro-focused ultrasound treatment sessions improves the efficacy of the skin rejuvenation process [15]. For example, in one study, Sasaki et al. used a 4 MHz 4.5 mm transducer. Following that, a 7 MHz 3.0 mm transducer was used to treat the neck and face regions. According to two blinded doctors, eight out of 10 evaluable patients demonstrated therapeutic benefit 90 days following therapy while nine subjects claimed improvement [16]. Another study by Fabi et al. investigated the effectiveness of MFU-V therapy at one or two treatment depths, the effect of changing the orientation and number of treatment lines, and the total energy applied during the treatment procedure. They discovered that 15 vertically oriented treatment lines with tissue depths of 4.5 mm and 3.0 mm significantly raised oppositional marionette lines and brows compared to 15 horizontally oriented treatment lines [17]. In general, the combination of treatment lines and increased energy at dual depths resulted in much more lifting in areas receiving treatment lines.

In research by Shome et al., Ulthera was used to treat 50 adult Indian individuals with sagging in their midface and lower face. The participants were treated with Ulthera 3.0 mm probes that targeted the deep dermis and 4.5 mm probes that targeted the superficial muscular aponeurotic system. Allergic responses and adverse effects, such as scarring and nerve/muscle malfunction, were assessed in all individuals. At 30 days, 60 days, three months, six months, and a year, the participants' and investigators' Global Aesthetic Improvement Scales scores were compared [18]. Photographs were gathered to provide a thorough facial analysis. In addition, a self-assessment questionnaire was given to the patients. Blinded evaluators noted improvement in the midface and lower face in 93% of patients after six months. The satisfaction of the patients with results was 85%. After a year, the very same results were sustained. This study found that employing focused ultrasound, which delivers treatment at a single focal depth, significant outcomes in the overall aesthetic improvement of drooping of the mid and lower face can be accomplished.

Oni et al. conducted research on lower face laxity to find out the effectiveness and safety of novel micro-focused ultrasound (MFU) equipment [19]. The study included participants who had MFU treatment for skin tightening. The primary end measure was improvements in lower-face skin laxity, as measured by improvements in jawline abnormalities, submental laxity, and marionette line. Subject Global Aesthetic Improvement Scale (SGAIS) and Investigator Global Aesthetic Improvement Scale (IGAIS) evaluations were collected from two blinded dermatologists who paired before and after treatment images.

After one session of MFU administration, 24 individuals were examined after a median of 4.3 months. According to the IGAIS, five patients (20.9%) showed improvement while 15 subjects (62.5%) showed no particular change. Worsening was assigned to four individuals (16.7%). According to the SGAIS, 11 people (45.9%) claimed an improvement while nine people (37.5%) showed no specific change. Additionally, a statistically important difference was observed between the participant’s and investigators' improvement rates. Investigators had a lower score (P =.006). There were no major side effects [19]. The comparatively high improvement percentage reported in the subjects' self-assessments contrasted with the images' investigators' evaluations. It showed that novel evaluation methods, other than photography, are needed to reflect what subjects feel but clinical investigators cannot see.

There are some drawbacks to this study. First, due to the high expense of this cosmetic operation, only a restricted number of participants were involved in the study. Second, there was no control group in this study. Other limitations include that assigning subjective and objective assessments were built on pre and post-treatment images, which was not a quantifiable technique for assessment of rejuvenation of the skin.

Suh et al. revealed that 63.6% of patients had clinical improvement two months after using a new MFU device with multiple 440 lines for the submentum and cheeks. In the same investigation, more collagen fibers in the lower dermis, as well as between fat layers, were seen histopathologically. Suh and colleagues recently proposed an improved procedure for applying another MFU device that included 200 to 300 lines per face in each treatment session and a total of three sessions set apart four weeks. At the three-month follow-up, researchers found that skin laxity was substantially improved and was improved slightly in 32.1%, 57.1%, and 10.7% of all 28 individuals, respectively, compared to baseline, with no serious side effects such as facial fat atrophy [20].

In a different study by Chang et al., a total of 25 individuals were enrolled. The face and neck were irradiated with MFU-V utilizing two separate transducers: One of 4 MHz, 4.5-mm focal depth, and the other with 7 MHz, 3.0-mm focal depth, totaling 800 lines. At 0, 90, and 180 days, the participants were assessed using a skin complexion analysis as well as a 3-dimensional imaging system. The mean brow height lift and the submental lift were measured. All 25 subjects finished treatment and had their 90-day and 180-day follow-up exams. Two of the twenty-five participants were men [21]. The average age of the patients was 53.3 years (ranging from 39.8 to 61.1 years). Skin laxity was studied using three variables: texture, wrinkles, and pores. Only the decline in mean wrinkles scores after 90 days was statistically related and significant (p=0.0222). At 90 days, there was a 0.47 mm brow lift (p=0.0165), however, at 180 days, there was a 0.12 mm decrease in brow height as compared to baseline (p=0.6494). A mean submental lift of 26.44 mm was seen after 90 days (p=0.0217). At 180 days, there was a mean 13.76 mm submental lift (p=0.243).

Kerscher M et al. conducted a study to investigate the efficacy of micro-focused ultrasound in conjunction with imaging in clinical research and daily practice. According to guidelines, 22 women with moderate-to-severe skin sagging along the submental region and jawline received a single MFU-V therapy. Short-term effects were assessed for up to three days following treatment, whereas long-term effects were measured for up to 24 weeks post-treatment. Under standardized settings, transepidermal water loss, flexibility, erythema, skin hydration, temperature, density, and skin thickness were measured. A standardized numeric visual analog scale was used to assess pain.

The study found that after MFU-V therapy, skin temperature stayed within a physiologic range and that there was no marked increase on Day 3. Transepidermal water loss, erythema levels, and hydration remained very steady across time, with no significant variations between short- and long-term measures compared to baseline. Gross and net elasticity values were considerably lowered (with values of P=0.0001 and P=0.003, respectively) after the application of MFU-V therapy alone at Week 4, continued by considerably increased values at Week 12 (P=0.046 and P=0.015) and Week 24 (P=0.049 and P=0.001). The edema caused by MFU-V therapy went away without causing any complications. All of the patients' pain subsided quickly, following treatment. During the 24-week follow-up phase, no side effects were reported. MFU-V therapy is well-accepted in this trial, and it has no effect on the epidermal barrier function and on skin physiology [22].

In a study by Chen et al., participants got one to three full-face therapies by the focused ultrasound device. To produce a single pass of microthermal coagulation zones, three transducers were utilized without the application of contemporary anesthetics (7.0 MHz, 3.0 mm focal depth; 7.0 MHz, 4.5 mm focal depth; 4.0 MHz, 4.5 mm focal depth). Two independent physicians evaluated standardized pictures recorded at baseline and at each follow-up. About six months after treatment, negative effects were examined. Patient questionnaires were also used to examine subjective pain and tolerance scores. A total of 68 therapy sessions were completed by 49 Chinese patients having skin types III-IV, with a mean age of 53.3. Transcutaneous focused ultrasound seems to be safe for noninvasive skin tightening of the face in Asians, according to study findings. The side effects were minor and only lasted a short time. Up to six months after therapy, no major long-term or delayed side effects were seen [23].

Besides, the first evidence of clinical efficacy of micro-focused ultrasound therapy in non-facial areas was published by Alster and Tanzi. Two-fold plane therapy with the 4-MHz 4.5-mm-depth and 7-MHz 3-mm-depth transducers was compared to single therapy with the 4-MHz 4.5-mm-depth transducer alone. The study was performed on 18 women on areas of the knees, arms, or medial thighs. Two blinded physician evaluators determined the global evaluation scores of skin lifting and tightening, which were evaluated on a quartile grading scale. All three body sites showed statistically considerable improvement at six months of examination, with the knees and arms showing more visible improvement than the thighs. The dual-plane therapy also helped to smooth skin texture, which could be linked to more superficial dermal collagen remodeling. When asked how satisfied they were with the treatment's clinical efficacy, 13 of 16 patients said they were very satisfied [24].

## Conclusions

MFU causes thermal tissue injury, resulting in microcoagulative zones that drive collagen neosynthesis and skin tightening. The lower face and neck, as well as non-facial areas, can yield promising outcomes. As a result, the use of MFU in cosmetic medicine is on the rise. Subsequently the effectiveness of MFU for facial rejuvenation, multiple independent scientists have successfully tested its application for tightening and lifting loose skin in various anatomic locations. Noninvasive skin raising of the upper arms, thighs, and knees is one of them. The efficacy of the MFU method is comparable to that of ablative or nonablative laser therapies, with minimal and temporary side effects. More research is required to govern the usage of MFU treatment for a larger range of clinical conditions.
